# Do coping mechanisms moderate the effect of stressful life events on depression and anxiety in young people? A case–control study from Latin America

**DOI:** 10.1136/bmjment-2024-301087

**Published:** 2025-01-09

**Authors:** Georgie Hudson, Catherine Fung, Diliniya Stanislaus Sureshkumar, Carlos Gómez-Restrepo, José Miguel Uribe-Restrepo, Karen Ariza-Salazar, Francisco Diez-Canseco, Liliana Hidalgo-Padilla, Mauricio Toyama, Luis Ignacio Brusco, Natividad Olivar, Santiago Lucchetti, Stefan Priebe, James B Kirkbride

**Affiliations:** 1 Division of Psychiatry, UCL, London, UK; 2 Unit for Social and Community Psychiatry, Queen Mary University of London, London, UK; 3 Department of Psychiatry and Mental Health, Pontificia Universidad Javeriana, Bogota, Colombia; 4 Department of Clinical Epidemiology and Biostatistics, Hospital Universitario San Ignacio, Bogota, Colombia; 5 CRONICAS Center of Excellence in Chronic Diseases, Universidad Peruana Cayetano Heredia, Lima, Peru; 6 Department of Psychiatry and Mental Health, University of Buenos Aires, Buenos Aires, Argentina; 7 Unit for Social and Community Psychiatry, East London NHS Foundation Trust, London, UK

**Keywords:** Child & adolescent psychiatry, Anxiety disorders, Depression & mood disorders, Data Interpretation, Statistical, Depression

## Abstract

**Background:**

Stressful life events (SLEs) are associated with increased risk of depression or anxiety. Coping mechanisms may moderate this relationship but little is known on this topic in young people or in Latin America.

**Aim:**

To investigate whether coping strategies predict odds of depression and/or anxiety and moderate the relationship between SLEs and depression and/or anxiety in young people in Peru, Lima and Bogotá.

**Method:**

Using case–control data from people aged 15–24, we used logistic regression to examine associations between coping mechanism, SLEs and caseness for depression or anxiety, adjusting for sociodemographic and socioeconomic factors. We included interaction terms to model whether this association varied depending on coping mechanisms (positive cognitive restructuring, problem focused, support seeking, distraction, avoidant).

**Results:**

We included 1437 cases and 965 controls. Cases reported less use of positive cognitive restructuring (OR 0.66; 95% CI 0.57 to 0.75) and problem-focused coping (OR 0.82; 95% CI 0.73 to 0.93), and more use of avoidance than controls (OR 1.33; 95% CI 1.19 to 1.50) in adjusted models. They had greater odds of reporting lifetime (OR 1.07; 95% CI 1.04 to 1.10) and past-year (OR 1.05; 95% CI 1.01 to 1.10) SLEs than controls. We found weak but consistent evidence of effect modification; the association between lifetime SLEs and case–control status was stronger in those who used less support seeking (p=0.09), problem-focused coping (p=0.08) or positive cognitive restructuring (p=0.09).

**Conclusions:**

Relationships between SLEs, coping mechanisms and depression/anxiety appear similar in these Latin American cities to other contexts. Active coping strategies may ameliorate the impact of SLEs on mental health of young people.

**WHAT IS ALREADY KNOWN ON THIS TOPIC**

- Stressful life events can increase the risk of experiencing mental health problems. By using certain coping mechanisms, this risk may be decreased, but coping mechanisms are not often looked at in young people, who are exposed to different events and may cope differently to adults. In addition, most of this research has been conducted in the Global West, so it is unclear if these relationships will be different here.

**WHAT THIS STUDY ADDS**

- We found that young people with symptoms of depression and/or anxiety in Latin America are less likely to use positive, active coping strategies and more likely to use avoidance as a coping strategy. Using these active coping strategies (support seeking, problem-focused coping and positive cognitive restructuring) appears to reduce the impact of stressful life events on mental health.

**HOW THIS STUDY MIGHT AFFECT RESEARCH, PRACTICE OR POLICY**

- By encouraging and teaching the use of support seeking, problem-focused coping and positive cognitive restructuring coping, we may be able to help prevent a decline in mental health after exposure to upsetting events. Further longitudinal research is needed to confirm any causal relationships between coping strategy and depression or anxiety.

## Introduction

 There are strong associations between stressful life events (SLEs) in childhood and the chances of developing depressive or anxiety symptoms or disorders.^1^ For example, a meta-analysis found that child maltreatment increased the risk of depressive or anxiety disorders by 2.48 and 1.68 times, respectively.^2^ Nonetheless, most studies of childhood trauma and mental health to date have been conducted in the Global North, including 89% of the studies in the above meta-analysis^2^; only 5% included people in Latin America. This meta-analysis found that in non-clinical population-level studies, all forms of child maltreatment (physical abuse, sexual abuse, emotional abuse, neglect and domestic violence) were associated with depressive disorders, and most with anxiety disorders. However, this meta-analysis did not consider SLEs beyond maltreatment, including parental death or experiencing a serious illness or injury.

It is possible that the relationship between SLEs and common mental disorders differs across continents and contexts. For example, exposure to childhood trauma and adversities has been reported to be higher in Latin America than other contexts.^3^ Reasons for this may include greater risk of exposure to poverty, poor education and housing, low employment rates or substance use.^4^ Due to more limited financial resources and mental healthcare investment, young people in Latin America rarely receive formal mental healthcare.^5^ Despite this, most young people appear to recover from depression within a year.^6^ It is unclear what traits may reduce mental health symptoms for these young people.

One possibility is that young people in these contexts use different types or levels of coping mechanisms. In a study from the Netherlands, self-blame, rumination and catastrophising were all associated with worse depressive symptoms in adolescents, while positive reappraisal appeared to be protective.^7^ They also found that the relationship between stress and depressive symptoms was stronger in those who employed coping strategies of self-blame or rumination.^7^ Other evidence suggests that active and problem-solving coping strategies positively impact mental health, whereas emotion-focused and passive (including distraction and avoidance) strategies have negative impacts.^8^ Despite this, coping strategies in young people have received little attention,^9^ and it is unclear to what degree different coping strategies moderate associations between SLEs and depression or anxiety in young people outside of the Global North.

We sought to clarify these issues in a sample of young people in three major cities in Latin America. Using these data, we have previously demonstrated that young people with depression or anxiety are more likely to experience SLEs.^10 11^ In this paper, we sought to address whether different forms of coping predict the odds of experiencing depression and/or anxiety, and whether these moderated the relationship between SLEs and odds of depression or anxiety.

We hypothesised that young people with depression and/or anxiety would have decreased odds of using active coping strategies (positive cognitive restructuring, support seeking and problem-focused coping) and increased odds of passive coping strategies (avoidance and distraction coping). We hypothesised that active coping strategies would lessen the association between SLEs (lifetime or past year) and depression and anxiety caseness, while greater use of passive coping strategies would increase this association.

## Methods

### Study design

We used data from the OLA Project^10 12^; a large, multisite study based in three study locations in Latin America: Buenos Aires (Argentina), Bogotá (Colombia) and Lima (Peru). Two groups of participants were recruited: those aged 15–16 and those aged 20–24.

### Sampling strategy and inclusion criteria

We recruited participants living in the most deprived half of administrative districts in each city. In Lima and Bogotá, we used the United Nations Development Programme’s Human Development Index (HDI^13^) to estimate the proportion of households in each district achieving basic living standards. In Buenos Aires, we used the Unsatisfied Basic Needs Index (NBI^14^) to estimate the proportion of households in each district experiencing unmet needs. The bottom 50% of districts in HDI or NBI rankings were selected for sample recruitment.

We recruited participants using convenience sampling. At each site, we aimed to recruit 340 young people aged 15–16 and 20–24 who met threshold criteria for depression or anxiety (see below; ie, cases) and 340 people without (ie, controls). For full details, see Gómez-Restrepo *et al.*^10^

### Participants

Participants had to be aged between 15 and 16 or 20 and 24 years when providing informed consent, be resident in an included district and have capacity to provide informed consent/assent. Exclusion criteria were diagnosis of psychosis, bipolar disorder, schizophrenia, or cognitive impairment, or illiteracy. Informed consent was obtained from all participants aged 18 or over, and for those under 18, assent was obtained alongside informed consent from their parent/guardian.

### Measures

All measures used self-reported questionnaires.

#### Outcome

All participants completed the Patient Health Questionnaire-8^15^ and the General Anxiety Disorder-7^16^ to screen for depression and anxiety, respectively. We defined cases as participants who scored 10 or more on either instrument, consistent with established cut-offs.^15 16^ Participants who did not meet these thresholds were defined as controls.

#### Exposures

An adapted version of Heubeck and O’Sullivan’s scale^17^ was used to measure the number of SLEs experienced, which captures whether participants have experienced any of 30 SLEs in the past year or more than 1 year ago. This included events such as the participant/someone close to them experiencing a serious illness or injury, changing schools, serious financial problems and bullying. We assessed two exposure measures: the number of SLEs experienced in the participant’s lifetime and the number of SLEs experienced in the past year.

#### Moderators

We assessed participants’ coping strategies using a version of the Children’s Coping Strategy Checklist, modified by Cline *et al*.^18^ This assessed how frequently participants employed different coping strategies when experiencing problems. The 26 items mapped onto five different coping strategies: positive cognitive restructuring, problem-focused coping, distraction, avoidance and support-seeking strategies. The scores for each coping strategy were generated by averaging items on each domain. Due to an oversight during data collection, one item was not included (‘I try to figure out why things like this happen’; problem-focused coping), reducing the number of items to 25.

#### Confounders

Confounders were selected based on prior research^7 9^ and theoretical knowledge. We constructed a directed acyclic graph (online supplemental figure 1) to model the hypothesised causal pathways and to identify the following relevant confounding variables:

1. Gender (male/female/other).
2. Age group (aged 15–16 or 20–24).
3. Socioeconomic status (SES) measured via three variables:
  - Number of people per bedroom.
  - Whether the participant had health insurance (yes/no/I don’t know). Types of health insurance differed across the three countries due to availability, but reflected a mix of public, social, police/armed forces and private health insurance policies.
  - Highest parental education of either caregiver (no formal education/primary/secondary/higher education).
4. Parental history of mental health treatment: The participant reported whether their parent(s) had received mental health treatment (one parent/both parents/neither parent).
5. Neighbourhood safety: We estimated mean scores from seven items related to neighbourhood safety from the Short Social Capital Assessment Tool,^19^ which was specifically designed for use in low-income countries, and validated for use in Latin America.^20^ Items were scored yes/no. One item was subsequently reverse coded so for all questions, one represented higher neighbourhood safety than zero.
6. Drug and alcohol use: Nine items from the Alcohol, Smoking and Substance Involvement Screening Test,^21^ designed for adolescents, were used to assess drug and alcohol use. Participants were asked how often they had used various illegal drugs in the last 3 months. The drug most frequently used was entered in analyses. Drug and alcohol use were included separately.
7. Perceived social support: The Multidimensional Scale of Perceived Social Support^22^ is a 12-item measure, with high scale reliability exceeding 0.90.^23^ Total perceived social support was estimated by averaging scores from all 12 items.

In this study, age, gender and SES were considered the most essential covariates to include in our models; however, all were important and included in all analyses. We considered including other variables as confounders such as parental drug use and attachment type; however, through discussion and a search of the literature it was decided that these variables were unlikely to be confounders between SLEs and anxiety/depression.

### Statistical analysis

#### Missing data

Participant responses of ‘I don’t know’ were recoded as missing data. We used multiple imputation by chained equations to impute missing covariate, moderator and exposure data. 20 datasets were imputed using logistic, linear, multinomial logistic and ordinal logistic regressions including auxiliary variables. Our primary analysis investigated the association between coping mechanism use and case–control status using the imputed dataset, combined using Rubin’s rule,^24^ which we checked against our complete case analysis results in a sensitivity analysis. See the online supplemental material for further information.

#### Analysis procedure

We summarised continuous variables by estimating the median and IQR, given evidence of non-normally distributed data using the Shapiro-Wilk test (online supplemental table 1). We compared cases and controls on their exposure and covariate values using the χ^2^ test for categorical data and Mann-Whitney U test for continuous data. We also used these tests to investigate differences between participants with and without missing data on exposure and confounding variables.

We used logistic regression to establish whether coping strategies and SLEs were associated with case–control status. First, we fitted univariable models to examine the unadjusted association between each exposure and covariate and case–control status. Second, we fitted multivariable regressions, adjusting for confounders, including other coping strategies. We present ORs and 95% CIs. Third, we included interaction terms in our regression models to model the effect modification of the association between SLEs (lifetime and past year) and case–control status by the level of each coping strategy employed. All confounding variables and other coping strategies were controlled for in these analyses. Statistical significance of the interactions was determined via inspection of whether Wald p values met the threshold of p<0.05. We visualised any putatively relevant continuous by continuous interactions graphically using marginal plots.

For all models, we standardised continuous variables (coping strategies, neighbourhood safety and perceived social support) to have a mean of 0 and SD of 1. ORs represented the change in odds of caseness associated with a one SD change in the exposure.

All analyses were performed using Stata V.17.

## Results

### Sample characteristics

We recruited 2402 participants, comprising 1437 cases (59.8%) and 965 controls (40.2%).

Compared with controls, cases were more likely to have experienced SLEs in their lifetime and past year; use lower levels of all five coping mechanisms; have higher levels of drug and alcohol use; have a parent in receipt of mental health treatment; perceive less social support; report lower neighbourhood safety; have health insurance; and have parents with higher levels of education (all p<0.001; table 1).

**Table 1:** Participant demographics and summary statistics

Characteristic	All participants (N=2402)	Controls (n=965)	Cases (n=1437)	Statistic
Number of lifetime SLEs experienced, median (IQR)	9 (6–13)	8 (5–11)	10 (7–13)	z=−10.7,p<0.001
Number of SLEs experienced in the past year, median (IQR)	2 (1–4)	2 (1–3)	3 (1–5)	z=−9.1,p<0.001
Frequency of coping strategy used, median (IQR)				
Positive cognitive restructuring	2.3 (2.0–2.8)	2.7 (2.2–3.0)	2.3 (1.8–2.7)	z=12.3,p<0.001
Problem-focused coping	2.6 (2.0–3.0)	2.8 (2.2–3.2)	2.4 (2.0–3.0)	z=10.0,p<0.001
Distraction strategies	2.0 (1.5–2.5)	2.0 (1.5–2.5)	2.0 (1.5–2.5)	z=3.9,p<0.001
Avoidance strategies	2.7 (2.3–3.0)	2.7 (2.3–3.0)	2.7 (2.3–3.0)	z=2.4,p=0.02
Support-seeking strategies	2.0 (1.5–2.5)	2.0 (1.5–2.8)	1.8 (1.3–2.3)	z=9.3,p<0.001
Gender, n (%)				
Male	815 (34.0)	428 (44.4)	387 (26.9)	χ^2^(2)=80.6,p<0.001
Female	1560 (65.0)	533 (55.2)	1027 (71.5)	
Other	24 (1.0)	4 (0.4)	20 (1.4)	
Missing	3 (0.1)	–	3 (0.2)	
Age group, n (%)				
Young group (15–16 years)	1080 (45.0)	435 (45.1)	645 (44.9)	χ^2^(1)=0.009,p=0.93
Older group (20–24 years)	1322 (55.0)	530 (54.9)	7892 (55.0)	
Age, median (IQR)				
Young group (15–16)	15 (15–16)	15 (15–16)	15 (15–16)	z=0.4,p=0.71
Older group (20–24)	21 (20–23)	21 (20–23)	21 (20–23)	z=−0.6,p=0.57
Centre, n (%)				
Argentina	621 (25.9)	280 (29.0)	341 (23.7)	χ^2^(2)=15.4,p<0.001
Colombia	965 (40.2)	344 (35.7)	621 (43.2)	
Peru	816 (34.0)	341 (35.3)	475 (33.1)	
Illicit drug use in the last 3 months, n (%)				
Not used	1964 (81.8)	848 (87.9)	1116 (77.7)	χ^2^(4)=40.4,p<0.001
Once or twice	182 (7.6)	47 (4.9)	135 (9.4)	
Monthly	66 (2.8)	19 (2.0)	47 (3.3)	
Weekly	77 (3.2)	18 (1.9)	59 (4.1)	
Every day/almost every day	99 (4.1)	29 (3.0)	70 (4.9)	
Missing	14 (0.6)	4 (0.4)	10 (0.7)	
Alcohol use in the last 3 months, n (%)				
Not used	1082 (45.1)	495 (51.3)	587 (40.9)	χ^2^(4)=37.0,p<0.001
Once or twice	756 (31.5)	297 (30.8)	459 (31.9)	
Monthly	302 (12.6)	89 (9.2)	213 (14.8)	
Weekly	234 (9.7)	75 (7.8)	159 (11.1)	
Every day/almost every day	23 (1.0)	6 (0.6)	17 (1.2)	
Missing	5 (0.2)	3 (0.3)	2 (0.1)	
Parent received mental health treatment, n (%)				
Neither parent	1532 (63.8)	667 (69.1)	865 (60.2)	χ^2^(2)=17.1,p<0.001
One parent	256 (10.7)	85 (8.8)	171 (11.9)	
Both parents	36 (1.5)	7 (0.7)	29 (2.0)	
Missing	578 (24.1)	206 (21.3)	372 (26.0)	
Ever experienced, n (%)				
Low mood	1298 (54.0)	302 (31.3)	996 (69.3)	χ^2^(1)=337.5,p<0.001
Anxiety	1115 (46.4)	228 (23.6)	887 (61.7)	χ^2^(1)=338.2,p<0.001
If ever experienced, currently experiencing, n (%)				
Low mood	769 (32.0)	89 (9.2)	680 (47.3)	χ^2^(1)=154.8,p<0.001
Anxiety	789 (32.9)	109 (11.3)	680 (47.3)	χ^2^(1)=115.6,p<0.001
If experienced mental health problems, ever received mental health treatment, n (%)				
Yes	380 (23.7)	82 (20.3)	298 (24.8)	χ^2^(1)=3.1,p=0.078
No	1232 (76.1)	321 (79.3)	911 (75.1)	
Missing	6 (0.3)	2 (0.5)	4 (0.3)	
Perceived social support, median (IQR)	4.9 (4.1–5.8)	5.3 (4.5–6.1)	4.7 (3.8–5.5)	z=12.5,p<0.001
Neighbourhood safety, median (IQR)	0.2 (0–0.3)	0.3 (0.1–0.4)	0.2 (0–0.3)	z=10.3,p<0.001
Highest parental education, n (%)				
No formal education	33 (1.4)	12 (1.2)	21 (1.5)	χ^2^(3)=17.9,p<0.001
Some primary education	306 (12.7)	139 (14.4)	167 (11.6)	
Some secondary education	1009 (42.0)	436 (45.2)	573 (39.9)	
Some higher education	955 (39.8)	335 (34.7)	620 (43.2)	
Missing	99 (4.1)	43 (4.5)	56 (3.9)	
Number of people per bedroom, median (IQR)	1.6 (1.3, 2.0)	1.7 (1.3–2.0)	1.5 (1.3–2.0)	z=1.1,p=0.28
Has health insurance, n (%)				
Yes	1676 (69.8)	635 (65.8)	10 341 (72.4)	χ^2^(1)=4.3,p=0.038
No	424 (17.7)	184 (19.1)	240 (16.7)	
Missing	302 (12.6)	146 (15.2)	156 (10.9)	

SLEstressful life event

### Missing data

Of the 2402 participants, 858 (35.7%) had some missing covariate or exposure data, although the percentage of item-level missing data was low (0.92%). Those with missing data were more likely to be in the younger age group; have lower drug use; use lower levels of positive cognitive restructuring and support-seeking strategies; and perceive less social support (all p<0.001; online supplemental table 2). Those with missing data were no more likely to be cases than controls (χ^2^(1)=2.2, p=0.14).

### Main effects of coping mechanisms and SLEs on depression and anxiety

In our univariable models, young people with depression or anxiety had decreased odds of using all forms of coping—positive cognitive restructuring (OR 0.60; 95% CI 0.55 to 0.65), problem-focused coping (OR 0.65; 95% CI 0.60 to 0.71), distraction strategies (OR 0.85; 95% CI 0.78 to 0.92), avoidance strategies (OR 0.91; 95% CI 0.84 to 0.99) and support-seeking strategies (OR 0.70; 95% CI 0.64 to 0.76)—compared with controls. After adjustment for covariates, the relationship between caseness and distraction (OR 1.07; 95% CI 0.96 to 1.18) and support-seeking strategies (OR 0.93; 95% CI 0.83 to 1.04) was no longer statistically significant, while the effect of avoidance strategies changed direction, whereby cases had increased odds of using this coping mechanism (OR 1.33; 95% CI 1.19 to 1.50). Cases remained less likely than controls to use positive cognitive restructuring (OR 0.66; 95% CI 0.57 to 0.75) and problem-focused coping strategies (OR 0.82; 95% CI 0.73 to 0.93) in multivariable models. Our adjusted logistic regression model following multiple imputation showed each additional lifetime (OR 1.07; 95% CI 1.04 to 1.04) and past-year (OR 1.05; 95% CI 1.01 to 1.10; table 2) SLE was associated with increased odds of depression and/or anxiety.

**Table 2:** Logistic regression model results for the association between SLEs, coping mechanisms, and depression or anxiety (20 imputations)

Characteristic	Univariable model			Multivariable model*		
	OR	95% CI		OR	95% CI	
		Low	High		Low	High
Number of SLEs experienced in the lifetime	1.11†	1.09	1.14	1.07†	1.04	1.10
Number of SLEs experienced in the past year	1.17†	1.13	1.21	1.05†	1.01	1.10
Positive cognitive restructuring (z-standardised)	0.60†	0.55	0.65	0.66†	0.57	0.75
Problem-focused coping (z-standardised)	0.65†	0.60	0.71	0.82†	0.73	0.93
Distraction strategies (z-standardised)	0.85†	0.78	0.92	1.07	0.96	1.18
Avoidance strategies (z-standardised)	0.91†	0.84	0.99	1.33†	1.19	1.50
Support-seeking strategies (z-standardised)	0.70†	0.64	0.76	0.93	0.83	1.04
Gender						
Male (Ref)	1			1		
Female	2.13†	1.08	2.54	2.07†	1.69	2.54
Other	5.54†	1.88	16.34	2.57	0.81	8.20
Age group						
Young group (15–16 years) (Ref)	1			1		
Older group (20–24 years)	1.01	0.86	1.19	0.91	0.74	1.12
Illicit drug use in the last 3 months						
Not used (Ref)	1			1		
Once or twice	2.18†	1.55	3.07	1.47	0.99	2.16
Monthly	1.88†	1.09	3.22	1.61	0.87	2.97
Weekly	2.51†	1.47	4.28	1.79	0.95	3.35
Every day/almost every day	1.84†	1.19	2.87	1.59	0.94	2.72
Alcohol use in the last 3 months						
Not used (Ref)	1			1		
Once or twice	1.30†	1.08	1.57	1.13	0.91	1.42
Monthly	2.02†	1.53	2.65	1.71†	1.24	2.37
Weekly	1.80†	1.33	2.42	1.75†	1.21	2.52
Every day/almost every day	2.39	0.94	6.12	2.79	0.95	8.15
Parent received mental health treatment						
No (Ref)	1			1		
One parent	1.55†	1.17	2.05	1.31	0.96	1.80
Both parents	2.73†	1.28	5.85	2.49†	1.06	5.84
Perceived social support (z-standardised)	0.57†	0.52	0.62	0.72†	0.64	0.80
Neighbourhood safety (z-standardised)	0.63†	0.57	0.69	0.75†	0.67	0.83
Highest parental education						
None	0.93	0.45	1.93	0.77	0.33	1.79
Primary	0.65†	0.50	0.84	0.70†	0.51	0.96
Secondary	0.71†	0.59	0.85	0.73†	0.59	0.91
Higher (Ref)	1			1		
Number of people per bedroom	1.01	0.93	1.10	1.06	0.96	1.18
Has health insurance						
No (Ref)	1			1		
Yes	1.29†	1.05	1.60	1.28	0.99	1.66

* Adjusted for all variables in the model.

† Indicates significantce at the level of p<0.05.

SLEstressful life event

We observed only minor differences between the analysis using the imputed dataset and complete case analysis (online supplemental table 3), suggesting that the missing data mechanisms underlying our dataset were at least missing at random and unrelated to variables not observed in this study.

### The relationship between SLEs and depression and anxiety for different coping strategies employed

**Figure 1:**
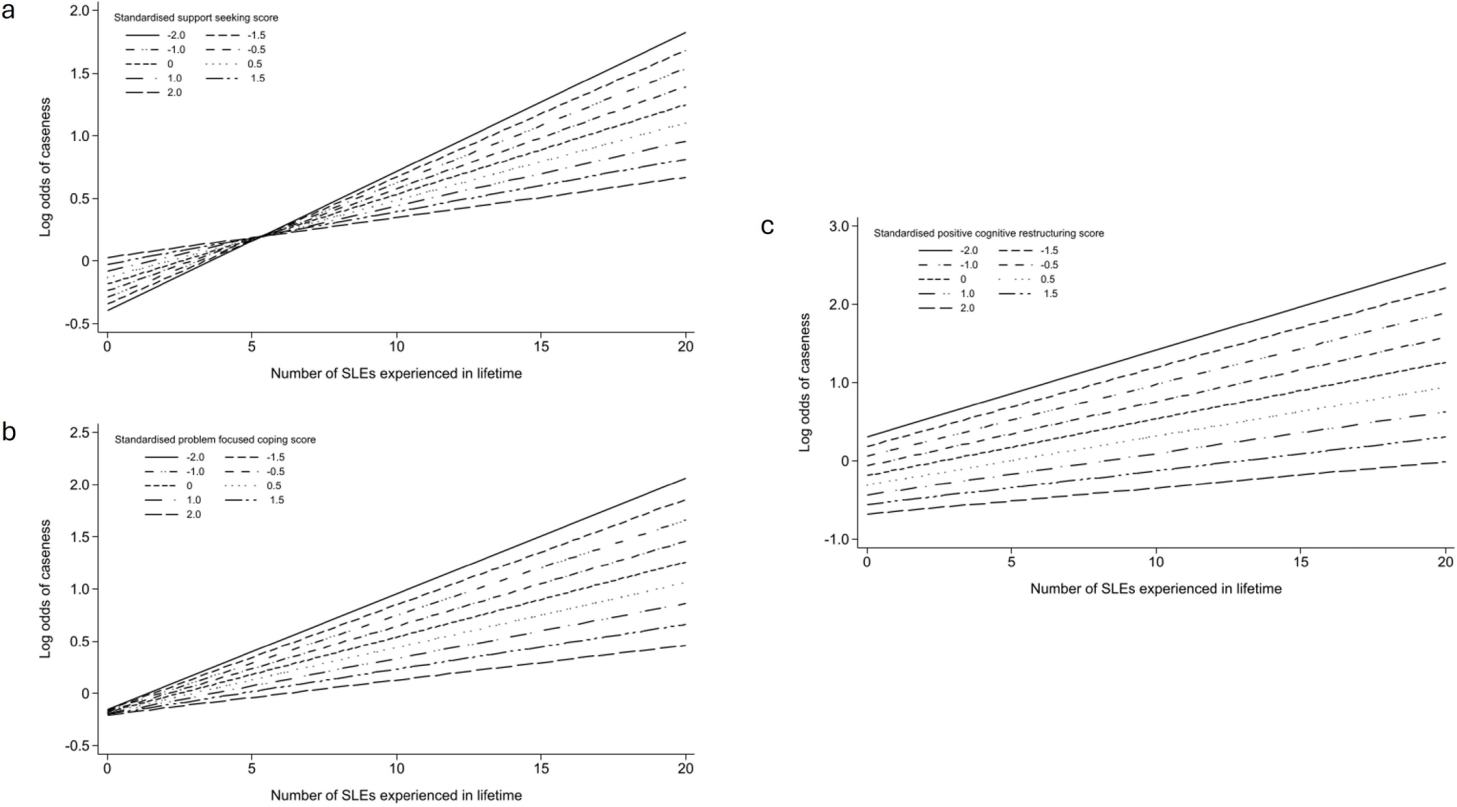
Marginal plot depicting the relationship between stressful life events (SLEs) and odds of depression/anxiety caseness, depending on the level of (**a**) support seeking, (**b**) problem-focused coping or (**c**) positive cognitive restructuring used.

We found weak but consistent trends to suggest that the relationship between lifetime SLEs and depression/anxiety was modified by active coping strategies; positive cognitive restructuring (p=0.09), problem-focused coping (p=0.08) and support seeking (p=0.09). We visualised these interactions in marginal plots (figure 1). These suggested that the relationship between increased odds of depression and/or anxiety and more lifetime SLEs was weaker for participants with greater use of support seeking, problem-focused coping and positive cognitive restructuring. There was no evidence of effect modification between lifetime SLEs and either passive coping strategy (distraction p=0.37, avoidance p=0.84) on the odds of depression and/or anxiety, and no evidence of effect modification with any coping style when only SLEs in the last year were considered (all p>0.35; online supplemental table 4).

## Discussion

### Principal findings

There is limited previous research investigating the impact of coping strategies on depression and/or anxiety, and whether coping can moderate the relationship between SLEs and anxiety and/or depression in young people, particularly in the Global South. When controlling for confounders, we found young people with depression and/or anxiety had decreased odds of using positive cognitive restructuring and problem-focused coping, and increased odds of using distraction strategies. Young people with depression and/or anxiety were more likely to report more SLEs. We found weak but consistent evidence that active coping strategies (positive cognitive restructuring, problem-focused coping and support seeking) buffered against the increased odds of depression and/or anxiety associated with experiencing more lifetime SLEs. No moderating effect was observed for more passive coping strategies (avoidance, distraction).

### Comparison with prior literature

Similar to Kraaij *et al*,^7^ we found the relationship between SLEs and depression was stronger for young people who used active coping strategies less frequently. We have shown that this relationship also exists for support-seeking strategies and when both depression and anxiety were included in our outcome definition. Additionally, it appears this relationship may generalise across settings, as our work in Latin America was consistent with that in the Netherlands.^7^ Although the interactions we observed between three active coping strategies and lifetime SLEs did not achieve conventional statistical significance (p=0.08), these consistent results suggest that active coping strategies may help buffer against the odds of developing anxiety or depression after experiencing SLEs.

In our sample, cases reported statistically significantly lower levels of perceived social support than controls. This may mean that it is harder for young people at risk of mental health problems to achieve the support seeking that may mitigate this risk. One method to increase the availability is via social support interventions, which appear to be beneficial. For example, children showed significantly reduced depressive symptoms following a 2-year social support intervention compared with no symptomatic difference in a control group who did not receive the intervention.^25^ Additionally, in our study, problem-focused coping appeared to moderate the relationship between lifetime SLEs and odds of depression/anxiety. A randomised controlled trial has shown that a 7-week mindfulness-based stress reduction programme for university students was associated with statistically higher levels of problem-focused coping, compared with a treatment as usual control group.^26^ Schools and colleges may be well placed to identify those at risk and implement interventions to improve their coping mechanisms or to provide them with the opportunity to seek support from an appropriate person.

We found no evidence of interactions between coping strategy and the number of SLEs experienced in the past year; only for the number experienced in the lifetime. There is evidence of a ‘sleeper effect’, whereby the impact of childhood trauma is not displayed until later in life.^27^ Young people exposed to more recent SLEs may not have had sufficient time to fully develop and implement their preferred coping strategy. Given the strong relationship we observed between SLEs in the past year and depression/anxiety, independent of lifetime SLEs, the immediate period after exposure to SLEs for young people may be a critical period for bonds to be developed to employ support-seeking strategies and minimise the risk of developing anxiety or depression.

### Strengths and limitations

Strengths of our study included a well-controlled case–control design, a large sample size, control for important covariates and low item-level missing data. We also employed multiple imputation techniques to account for any missing data, and results from our imputed sample were consistent with those from our complete case analysis. We included exposure, outcome, covariate and several auxiliary variables when performing the multiple imputation. The imputation approach we used assumes the data are at least missing at random. It is plausible that reasons for participants having missing data on exposure to SLEs were related to their unobserved exposure status. For example, participants may not have wanted to reveal they had experienced traumatic events if they were unsure this information would be treated confidentially. However, since other variables that predicted both missingness and exposure status were included in the imputation model, this increases the plausibility that the data were missing at random. Additionally, both SLE exposure and covariates had very low levels of missing data (except for parental mental health treatment), meaning any systematic differences in missingness are unlikely to have impacted the results. Therefore, the assumption of the data missing at random appears likely to have been met.

We also note several potential limitations which mean that causality cannot be inferred. First, case–control studies are subject to the possibility of recall bias. Recall bias may have acted differentially, if depressed or anxious participants were more or less likely to have recalled negative life events than control participants; there is evidence that people with common mental disorders under-report levels of childhood adversity.^28^

A second related issue is that we could not establish the temporal order between SLEs, coping strategies and caseness. Due to the case–control design, we were not able to infer any causal relationships between SLEs, coping strategies, and depression and/or anxiety. It is possible that coping strategies are related to cognitive biases that are influenced by symptoms of depression or anxiety; further longitudinal evidence is required to tease out the directionality of these relationships. Nonetheless, we hypothesised that coping strategies would moderate the association between SLEs and mental health, and found stronger support for this in relation to lifetime SLEs, consistent with longitudinal evidence on this topic.^29^ It has also been suggested that coping mechanisms may act as a mediator of the relationship between SLEs and psychopathology^30^; however, we could not investigate this given our study design.

Although we recruited a large sample, our study was likely to have been underpowered to detect statistical interactions between lifetime SLEs and coping strategies at the observed coefficient effect sizes. Although our observed p values for interactions between active coping strategies and lifetime SLEs were just outside of conventional statistical significance, these trends were consistent, and the buffering effects in the hypothesised direction. This lends credence to the possibility that active coping strategies can ameliorate the potential harmful effects of SLEs on mental health in young people, and warrants replication in larger longitudinal studies in diverse settings.

## Conclusions

There was some evidence to suggest that active coping strategies moderated the odds of depression/anxiety for young people who had experienced SLEs in three deprived Latin American settings. The evidence was strongest for the impact of problem-focused strategies in buffering the impact of SLEs on mental health, but this relationship may exist for positive cognitive restructuring and support seeking as well. Schools and community organisations may be well placed to offer interventions to young people at risk of experiencing mental health problems after exposure to SLEs to improve their coping mechanism and reduce their risk. However, due to the case–control design, causality cannot be inferred. Further longitudinal research is needed to confirm the existence of a moderating effect of coping mechanisms, particularly for young people living in deprived contexts outside the Global North.

## supplementary material

10.1136/bmjment-2024-301087 online supplemental file 1

## Data Availability

Data are available upon reasonable request.
