# Supplementary material for: Do coping mechanisms moderate the effect of stressful life events on depression and anxiety in young people? A case–control study from Latin America
Source: BMJ Ment Health. 2025 Jan 9;28(1):e301087. doi: 10.1136/bmjment-2024-301087 (PMC11751918; doi:10.1136/bmjment-2024-301087)
Supplement: online supplemental file 1 [file bmjment-28-1-s001.docx]

**Further information on missing data analysis procedure**

Multiple imputation by chained equations (MICE) operates under the assumption that the data are either missing at random or missing completely at random, i.e., there were no systematic differences between missing and observed values after observed data were taken into account (1). For this reason, we included auxiliary variables in our imputation models to provide information about missing values, and increase the plausibility data were at least missing at random. Included auxiliary variables were: 10 items from the Connor-Davidson Resilience Scale (2), a binary measure of whether the participant owned a smartphone, age, case-control status, and complete item-level data on SLEs.

For measures of SLEs, coping, neighbourhood safety, and perceived social support, data were imputed at the item level and then aggregated using passive imputation to create a total score. Estimates were combined across the 20 imputed datasets.

**References**

1. Sterne JAC, White IR, Carlin JB, Spratt M, Royston P, Kenward MG, et al. Multiple imputation for missing data in epidemiological and clinical research: Potential and pitfalls. Vol. 339, BMJ (Online). 2009. p. 157–60.

2. Connor KM, Davidson JRT. Development of a new resilience scale: The Connor-Davidson Resilience Scale (CD-RISC). Depress Anxiety. 2003 Sep;18(2):76–82.

| **Supplementary Table 1 – Shapiro-Wilk test for normal distribution results** | | |
| --- | --- | --- |
| **Variable** | **W** | **P-Value** |
| Positive cognitive restructuring | 1.00 | *p* < .001 |
| Problem focused coping | 0.99 | *p* < .001 |
| Distraction strategies | 0.98 | *p* < .001 |
| Avoidance strategies | 1.00 | *p* = .022 |
| Support seeking strategies | 0.98 | *p* < .001 |
| Number of SLEs experienced in lifetime | 0.99 | *p* < .001 |
| Number of SLEs experienced in the last year | 0.92 | *p* < .001 |
| Perceived social support | 0.98 | *p* < .001 |
| Neighbourhood safety | 0.99 | *p* < .001 |
| Number of people per bedroom | 0.73 | *p* < .001 |
| Age for younger age group | 0.98 | *p* < .001 |
| Age for older age group | 0.99 | *p* < .001 |

| **Supplementary Table 2 – Distribution of participant demographics and summary statistics, by complete data status.** | | | | |
| --- | --- | --- | --- | --- |
| **Characteristic** | | **Incomplete Data**  **(n = 858)** | **Complete Case**  **(n = 1,544)** | **Statistic** |
| Gender, n (%)* | |  |  |  |
|  | Male | 328 (38.3) | 487 (31.5%) | χ^2^ (2) = 13.1,  *p* = .001 |
|  | Female | 516 (60.1%) | 1044 (67.6%) |  |
|  | Other | 11 (1.3%) | 13 (0.8%) |  |
|  | Missing | 3 (0.4%) | — |  |
| Age group, n (%)* | |  |  |  |
|  | Young group (15-16 years) | 469 (54.7%) | 611 (39.6%) | χ^2^ (1) = 50.7,  *p* < .001 |
|  | Older group (20-24 years) | 389 (45.3%) | 933 (60.4%) |  |
| Centre, n (%)* | |  |  |  |
|  | Argentina | 250 (29.1%) | 371 (24.0%) | χ^2^ (2) = 7.6,  *p* = .02 |
|  | Colombia | 332 (38.7%) | 633 (41.0%) |  |
|  | Peru | 276 (32.2%) | 540 (35.0%) |  |
| Case status, n (%) | |  |  |  |
|  | Case | 530 (61.8%) | 907 (58.7 %) | χ^2^ (1) = 2.1,  *p* = .15 |
|  | Control | 328 (38.2%) | 637 (41.3 %) |  |
| Frequency of coping strategy used, median (IQR) | |  |  |  |
|  | Positive cognitive restructuring, median (IQR)* | 2.3 (2.0, 2.8) | 2.5 (2.0, 3.0) | *z* = -3.8,  *p* < .001 |
|  | Problem focused coping, median (IQR)* | 2.4 (2.0, 3.0) | 2.6 (2.0, 3.0) | *z* = -4.5,  *p* < .001 |
|  | Distraction strategies, median (IQR) | 2.0 (1.5, 2.5) | 2.0 (1.5, 2.5) | *z* = -1.2,  *p* = .22 |
|  | Avoidance strategies, median (IQR) | 2.7 (2.3, 3.0) | 2.7 (2.3, 3.2) | *z* = -1.7,  *p* = 0.095 |
|  | Support seeking strategies, median (IQR)* | 1.8 (1.3, 2.3) | 2.0 (1.5, 2.5) | *z* = -5.4,  *p* < .001 |
| Number of SLEs experienced in lifetime, median (IQR)* | | 10 (7, 13) | 9 (6, 12) | *z =* 2.6, *p* = 0.01 |
| Number of SLEs experienced in last year, median (IQR)* | | 3 (1, 5) | 2 (1, 4) | *z =* 2.6, *p* = 0.01 |
| Illicit drug use in last three months, n (%) | |  |  |  |
|  | Not used | 691 (80.5%) | 1273 (82.5%) | χ^2^ (4) = 5.2,  *p* = 0.27 |
|  | Once or twice | 57 (6.6%) | 125 (8.1%) |  |
|  | Monthly | 22 (2.6%) | 44 (2.9%) |  |
|  | Weekly | 34 (4.0%) | 43 (2.8%) |  |
|  | Every/almost every day | 40 (4.7%) | 59 (3.8%) |  |
|  | Missing | 14 (1.6%) | — |  |
| Alcohol use in last three months, n (%) | |  |  |  |
|  | Not used | 395 (46.0%) | 687 (44.5%) | χ^2^ (4) = 6.0,  *p* = 0.20 |
|  | Once or twice | 273 (31.8%) | 483 (31.3%) |  |
|  | Monthly | 95 (11.1%) | 207 (13.4%) |  |
|  | Weekly | 78 (9.1%) | 156 (10.1%) |  |
|  | Every/almost every day | 12 (1.4%) | 11 (0.7%) |  |
|  | Missing | 5 (0.6%) | — |  |
| Parent received mental health treatment, n (%) | |  |  |  |
|  | No | 237 (27.6%) | 1295 (83.9%) | χ^2^ (2) = 0.5,  *p* = 0.77 |
|  | One parent | 39 (4.6%) | 217 (14.1%) |  |
|  | Both parents | 4 (0.5%) | 32 (2.1%) |  |
|  | Missing | 578 (67.5%) | — |  |
| Perceived social support, median (IQR)* | | 4.8 (3.9, 5.6) | 5.0 (4.2, 5.8) | *z* = -5.1,  *p* < .001 |
| Neighbourhood safety (median, IQR) | | 0.2 (0, 0.3) | 0.2 (0, 0.4) | *z* = -1.4,  *p* = 0.17 |
| Number of people per bedroom, median (IQR) | | 1.7 (1.3, 2.0) | 1.5 (1.3, 2.0) | *z* = 0.3,  *p* = 0.77 |
| Has health insurance, n (%) | |  |  |  |
|  | Yes | 440 (51.3%) | 1236 (80.1%) | χ^2^ (1) = 0.21,  *p* = 0.65 |
|  | No | 116 (13.5%) | 308 (20.0%) |  |
|  | Missing | 302 (35.2%) | — |  |
| * Indicates significant at the level of *p* < .05. | | | | |

| **Supplementary Table 3 –Logistic regression model results for the complete case analysis association between SLEs, coping mechanisms, and depression or anxiety** | | | | | | | |
| --- | --- | --- | --- | --- | --- | --- | --- |
| **Characteristic** | | **Univariable Model** | | | **Multivariable Model** | | |
|  |  | Odds Ratio | 95 % CI | | Odds Ratio | 95% CI | |
|  |  |  | Low | High |  | Low | High |
| Number of SLEs experienced in lifetime | | 1.10* | 1.08 | 1.13 | 1.06* | 1.03 | 1.10 |
| Number of SLEs experienced in last year | | 1.16* | 1.11 | 1.22 | 1.08* | 1.02 | 1.14 |
| Positive cognitive restructuring (z-standardised) | | 0.59* | 0.53 | 0.66 | 0.62* | 0.52 | 0.73 |
| Problem focused coping (z-standardised) | | 0.63* | 0.57 | 0.70 | 0.77* | 0.65 | 0.89 |
| Distraction strategies (z-standardised) | | 0.89* | 0.80 | 0.98 | 1.12 | 0.99 | 1.27 |
| Avoidance strategies (z-standardised) | | 0.93 | 0.84 | 1.03 | 1.41* | 1.22 | 1.63 |
| Support seeking strategies (z-standardised) | | 0.74* | 0.67 | 0.83 | 1.01 | 0.88 | 1.15 |
| Gender | |  |  |  |  |  |  |
|  | Male (Ref) | 1 |  |  |  |  |  |
|  | Female | 2.02* | 1.62 | 2.51 | 1.09* | 1.47 | 2.46 |
|  | Other | 6.25* | 1.37 | 28.48 | 2.97 | 0.59 | 14.77 |
| Age group | |  |  |  |  |  |  |
|  | Young group (15-16 years) (Ref) | 1 |  |  | 1 |  |  |
|  | Older group (20-24 years) | 1.09 | 0.89 | 1.34 | 1.01 | 0.78 | 1.31 |
| Illicit drug use in last three months | |  |  |  |  |  |  |
|  | Not used (Ref) | 1 |  |  | 1 |  |  |
|  | Once or twice | 2.13* | 1.41 | 3.21 | 1.47 | 0.92 | 2.33 |
|  | Monthly | 1.90 | 0.98 | 3.66 | 1.56 | 0.74 | 3.31 |
|  | Weekly | 2.31* | 1.16 | 4.63 | 1.19 | 0.53 | 2.68 |
|  | Every/almost every day | 2.33* | 1.29 | 4.24 | 1.58 | 0.79 | 3.13 |
| Alcohol use in last three months | |  |  |  |  |  |  |
|  | Not used (Ref) | 1 |  |  | 1 |  |  |
|  | Once or twice | 1.30* | 1.03 | 1.64 | 1.12 | 0.85 | 1.47 |
|  | Monthly | 2.02* | 1.45 | 2.81 | 1.54* | 1.05 | 2.28 |
|  | Weekly | 2.16* | 1.48 | 3.14 | 1.90* | 1.20 | 3.00 |
|  | Every/almost every day | 2.41 | 0.63 | 9.15 | 1.68 | 0.39 | 7.17 |
| Parent received mental health treatment | |  |  |  |  |  |  |
|  | No (Ref) | 1 |  |  | 1 |  |  |
|  | One parent | 1.53* | 1.13 | 2.07 | 1.28 | 0.91 | 1.79 |
|  | Both parents | 3.29* | 1.35 | 8.05 | 2.94* | 1.12 | 7.72 |
| Perceived social support (z-standardised) | | 0.59* | 0.52 | 0.66 | 0.71* | 0.61 | 0.81 |
| Neighbourhood safety (z-standardised) | | 0.68* | 0.61 | 0.75 | 0.80* | 0.71 | 0.09 |
| Highest parental education | |  |  |  |  |  |  |
|  | None | 0.66 | 0.26 | 1.64 | 0.51 | 0.17 | 1.49 |
|  | Primary | 0.65* | 0.47 | 0.89 | 0.74 | 0.51 | 1.07 |
|  | Secondary | 0.79* | 0.63 | 0.98 | 0.85 | 0.66 | 1.10 |
|  | Higher (Ref) | 1 |  |  | 1 |  |  |
| Number of people per bedroom | | 1.00 | 0.90 | 1.12 | 1.06 | 0.93 | 1.20 |
| Has health insurance | |  |  |  |  |  |  |
|  | No (Ref) | 1 |  |  | 1 |  |  |
|  | Yes | 1.20 | 0.93 | 1.54 | 1.18 | 0.87 | 1.59 |
| * Indicates significant at the level of p < .05. | | | | | | | |

| **Supplementary Table 4 – Odds ratios and Wald *p*-values for imputed models investigating effect modification of coping strategy on the relationship between SLEs and odds of depression and/or anxiety** | | |
| --- | --- | --- |
| **Coping strategy** | **Number of SLEs experienced in lifetime** | **Number of SLEs experienced in previous year** |
| Positive cognitive restructuring | *p* = .086  OR: 0.98 (95% CIs: 0.96-1.00) | *p* = .71  OR: 1.01 (95% CIs: 0.97-1.05) |
| Problem-focused coping | *p* = .078  OR: 0.98 (95% CIs: 0.96-1.00) | *p* = .36  OR: 1.02 (95% CIs: 0.98-1.06) |
| Distraction strategies | *p* = .37  OR: 0.99 (95% CIs: 0.97-1.01) | *p* = .62  OR: 0.99 (95% CIs: 0.96-1.03) |
| Avoidance strategies | *p* = .84  OR: 1.00 (95% CIs: 0.98-1.02) | *p* = .74  OR: 1.01 (95% CIs: 0.97-1.04) |
| Support seeking strategies | *p* = .086  OR: 0.98, 95% CIs: 0.96-1.00) | *p* = .60  OR: 1.01 (95% CIs: 0.97-1.05) |
| *Both coping strategy and number of SLEs are continuous variables and therefore the odds ratios are for this interaction term between two continuous variables.* | | |

**
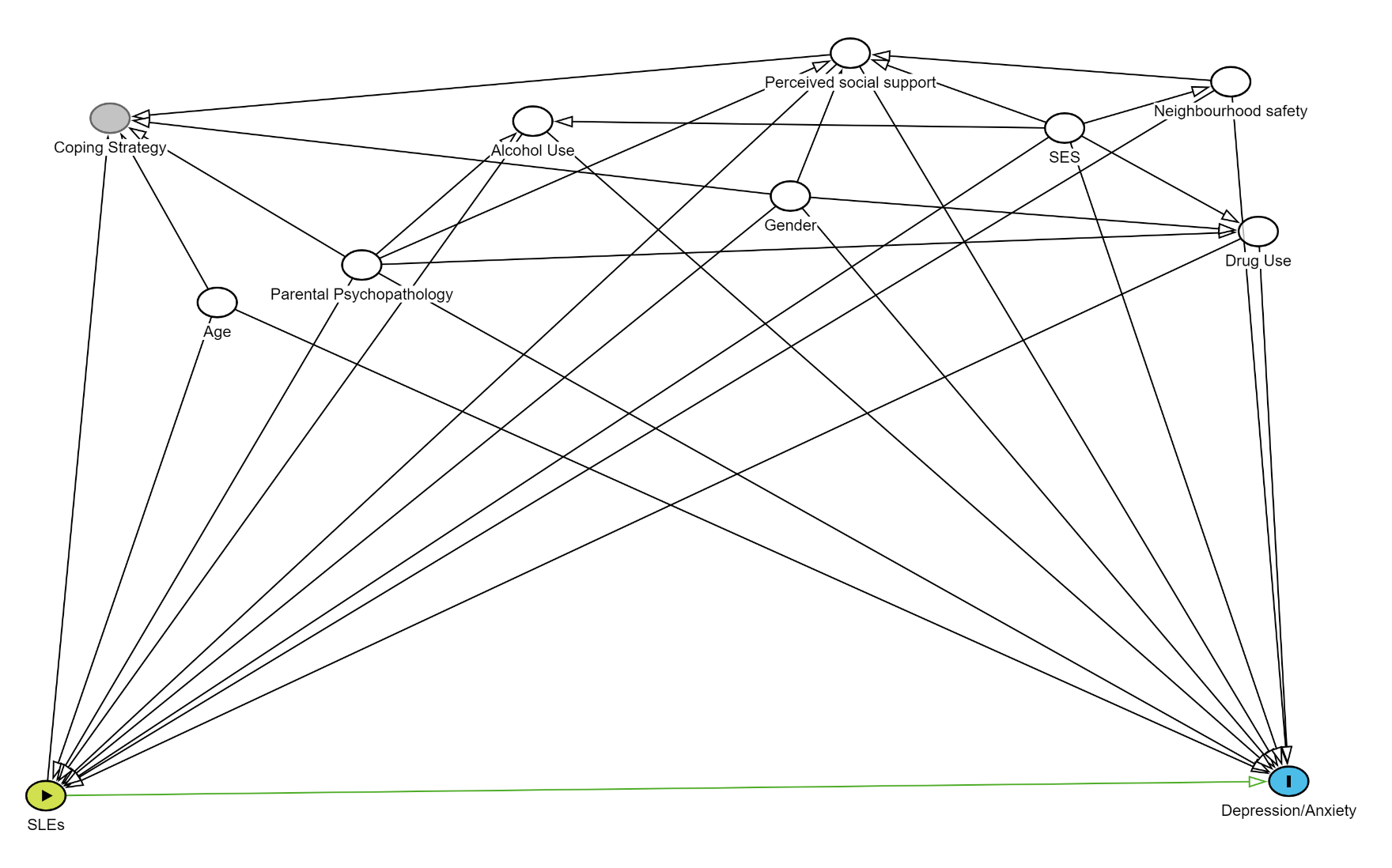
Supplementary Figure 1. Directed Acyclic Graph (DAG) used to model hypothesised causal relationships and identify confounders of the association between SLEs and depression/anxiety.**

**Figure legend:** This DAG was constructed using [www.daggity.net](http://www.daggity.net). The green circle with triangle is the exposure (stressful life events), and the blue circle with “I” is the outcome variable (depression and/or anxiety). The green line shows the direct causal path between exposure and outcome. The observed confounders in the dataset impact both SLEs and depression and/or anxiety, and were controlled for. Variables in grey do not need to be controlled for. Some of the confounders and life events also impact on coping strategy, which in turn is hypothesised to moderate the effect of life events on depression and anxiety.

Acronyms:
SLEs: Stressful life events
SES: Socioeconomic status
